# Adverse trends of cardiovascular risk factors among low risk populations (1983-1994) - a cohort study of workers and farmers in Guangzhou, China

**DOI:** 10.1186/1471-2458-11-931

**Published:** 2011-12-14

**Authors:** Xiaoqing Liu, Jinzhuang Mai, Xuxu Rao, Qiling Zhuo, Chengye Guo, Xiangmin Gao, Yong Wu, Mulan Deng, Shuguang Lin

**Affiliations:** 1 Guangdong Cardiovascular Institute, Guangdong General Hospital, Guangdong Academy of Medical Science, 96# Dongchuan Road, Guangzhou 510100, Peoples Republic of China

## Abstract

**Background:**

The levels and trends of cardiovascular risk factors vary greatly throughout China. We examine 10-year trends of cardiovascular risk factors (1983-1994) and the factors related to these trends among low-risk cohorts of workers and farmers in Guangzhou, China.

**Methods:**

This is a cohort study of 3,131 workers and 3,493 farmers aged 25-64 years at baseline with 10 years of follow-up. We performed a longitudinal analysis to account for the aging of the cohorts and the repeated measures of the same individual.

**Results:**

At baseline the prevalence of overweight (including obese) ranged from 1.0% to 11.8%, hypertension ranged from 3.8% to 10.5%, and mean serum total cholesterol (TC) ranged from 155.4 mg/dl to 187.2 mg/dl. Although prevalence of smoking declined, blood pressure levels and body mass index (BMI) increased significantly, and lipid profiles changed unfavorably during the 10-year follow-ups. The prevalence of hypertension increased from 5.0 percentage points (female farmers) to 12.3 percentage points (male farmers). Mean TC increased significantly (e.g., +22.8 mg/dl and +17.0 mg/dl in male and female farmers, respectively). In the longitudinal data analyses, increase in BMI was associated with increase in blood pressure levels and TC. Significant adverse trends of risk factors persisted after adjustment for aging, education, BMI, smoking, and alcohol intake.

**Conclusion:**

Urgent action is needed to prevent and reverse the unhealthy trends occurring among these low risk Chinese workers and farmers.

## Background

China's society and economy have been developing rapidly during the past 30 years. Although this growth has resulted in a marked increase in the standard of living, the health of Chinese population overall is becoming "westernized", characterized by increasing prevalence of hypertension, diabetes, and cardiovascular disease [1]. Levels and trends of cardiovascular risk factors as well as cardiovascular morbidity and mortality varied greatly throughout China [2-6]. Prevalence of hypertension and serum total cholesterol (TC) level was much higher in north China than in south China [4,5]. Surveillance data from Sino-MONICA project showed that there was an up to 33-fold difference in the incidence of coronary heart disease among men living in 17 different areas of the country during the late 1980s [2]. From 1987 through 1993, the incidence and mortality of cardiovascular disease increased in some areas, whereas it decreased in others [2]. In Beijing, blood pressure levels in the populations have been reported inconsistently as increased [7], unchanged, or somewhat decreased [6,8] from 1980s to 1990s. TC levels have increased [8] or decreased [9] in some cases as well. In Shanghai, mean blood pressure changed little, whereas TC markedly increased [6].

Guangzhou is a coastal city in the southeast corner of China. The people who live in Guangzhou are much leaner, with a body mass index (BMI) of around 20-21 kg/m^2 ^[10]. The national surveys [11,12] conducted during 1980s and 1990s showed that the prevalence of hypertension in Guangdong province, where Guangzhou is located, was less than 10% in adults. The prevalence was among the lowest in the country and much lower than those among various populations in the Asia-Pacific region [13], or in the world [14] including sub-Saharan Africa [15]. In 1984, coronary heart disease mortality among those aged 35-74 years in Guangzhou was one-third of that in Beijing and one-tenth (men) to one-fifth (women) of those in North America and Australia [16].

Guangzhou has developed faster, and its economy has grown more quickly than most other areas in China since 1980s. The purpose of this study is to examine the 10-year trends of cardiovascular risks (1983-1994) and the factors related to these trends among the low-risk cohorts of workers and farmers in Guangzhou, China.

## Methods

### Study cohorts

In 1981, a joint research program, the Collaborative Study of Cardiovascular and Cardiopulmonary Epidemiology, was initiated between the United State of American (USA) and the People's Republic of China (PRC) under the USA-PRC Cooperation in Science and Technology [17]. Cohorts of Chinese populations were established from four sites: two in northern China (urban and rural Beijing) and two in southern China (urban and rural Guangzhou). The study protocol was developed jointly by the scientists from the U.S. and the PRC. Field investigators were trained and certified before the data collections.

In urban Guangzhou, the participants were clustered samples from a factory--the Guangzhou Shipyard Company. All persons, primarily manual labor workers, aged 25-64 years from 8 of the 25 workshops were recruited. The response rates were 84% and 91% for males and females, respectively. The rural participants were from Dashi Township of Panyu County, 20 km south of Guangzhou City. Of the 21 village clusters, 14 were sampled. All persons, primarily farmers, aged 25-64 years were recruited. The response rates were 91% and 94% among males and females, respectively. The study was approved by the Science and Ethics Committee of the Guangdong General Hospital, the executive office of Guangzhou Shipyard Company and the municipal office of Dashi Township.

Baseline examinations were conducted for half of the cohort during 1983 and the other half during 1984. Follow-up re-examinations were conducted during 1987-1988 (4 years after baseline) and again during 1993-1994 (10 years after baseline). All examinations were performed during September and October. At baseline, 3,437 workers and 3,629 farmers completed the questionnaire interviews and physical examinations. Among them, 3,131 workers (1,564 males and 1,567 females) and 3,493 farmers (1,521 males and 1,972 females) had at least one re-examination and were included in this analysis. Excluding 91 persons who died before the first re-examination, the retention of the surviving cohort was 92% for workers and 98% for farmers.

### Data collection

Demographic, medical, behavioral, and lifestyle data were collected. Participants were asked if they ever attended school, graduated from primary school, middle school, high school, or college. In addition, the participant was asked if he or she was current smoker and the average number of cigarettes smoked per day. A small percentage of farmers were pipe smokers or smoked hand-made cigarettes. The equivalent number of cigarettes smoked per day was estimated by using the monthly consumption of tobacco leaves (about 1 g of tobacco per commercial cigarette). Daily alcohol intake was calculated by summing the intakes from various alcoholic beverages in the past week. The participants were asked to report the amount of drinking for each type of alcoholic beverage including beer, grape (or other fruits) wine, Huangjiu ("yellow wine" brewed from grains), rice wine, and liquor/spirit. The alcohol content was 3.6% for beer, 12% for grape wine, 12% for Huangjiu, 26% for rice wine, and 47% for liquor/spirit. Body weight was measured by using a spring scale, with participants in light clothing without shoes. Height was measured by using a vertical ruler. BMI was calculated as weight in kilogram (kg) divided by height in squared meter (m^2^). Overweight was defined as having a BMI of 25 kg/m^2 ^or higher and obesity was defined as BMI of 30 kg/m^2 ^or higher. Three systolic (SBP) and fifth-phase diastolic blood pressures (DBP) measurements were taken on the right arm with a mercury sphygmomanometer and the average of the three readings was used in the analysis. Hypertension was defined as SBP ≥ 140 mmHg and/or DBP ≥ 90 mmHg or on antihypertensive medication.

Participants were asked to fast for at least 12 h before blood samples were drawn. Some participants either did not fast for at least 12 h or they declined to have blood drawn. Baseline and at least one follow-up lipid measures were performed in 2,118 workers (1,072 males and 1,046 females) and 2,241 farmers (1,059 males and 1,182 females).

Blood lipids were measured and standardized according to the Lipid Standardization Program of the U.S. Centers for Disease Control and Prevention (CDC) and followed a quality control program using samples prepared by the CDC. The mean percentage biases based on means pooled TC measured, less CDC reference mean, was consistently less than 1% for both high and low pools. No significant laboratory drift was detected during 10 years [9].

At baseline and the first follow-up examination, the lipids were analyzed by using fresh blood samples. At the second follow-up examination (1993-1994), serum samples were frozen and analyzed 6 months later. The mean value from frozen samples was on average 2.2 mg/dl higher than that from fresh samples evaluated from 124 persons [9]. Hence, we performed additional data analyses adjusting for these small shifts in the 1993-1994 individual TC values.

Hyperlipidemia was defined as having a TC ≥220 mg/dl and/or LDL-C ≥160 mg/dl.

### Statistical methods

The student's *t*-test and Chi-square test (Fisher's exact test if appropriate) was used to compare mean and proportion between workers and farmers within the same gender. The longitudinal analyses were performed using the Generalized Estimating Equation (GEE) method [18] to examine the time trends of various measures (e.g., blood pressure, BMI, lipids) and factors related to these changes, accounting for the effect of aging and the correlations of repeated measures on the same person. The model for each gender-occupation group is as follows:

$$Y_{it}=\beta_{0}+\beta_{1}t+\beta_{2}X_{it}+\beta_{3}Z_{i0}+\beta_{4}\Delta Z_{it}+e_{it}$$

Where for *t *= 0,4,10, Y_*it *_is the dependent variable (e.g., SBP) for the *i*th person at time *t*; X_*it *_is a time-dependent covariate for the *i*th person at time *t *(i.e., age, use of antihypertensive medication); *Z*_*i0 *_is the baseline value of a time-independent covariate (i.e., baseline education, BMI, number of cigarettes smoked per day, and alcohol intake per day); Δ*Z*_*it *_= Z_*it*_-Z_*i0*_, that is the change in a covariate (i.e., BMI, number of cigarettes smoked per day, and alcohol intake per day) between time *t *and baseline for the *i*th person; and *e*_*it *_is the error term.

The coefficient *β*_*1 *_measures the average annual change in a dependent variable (e.g., SBP) adjusting for all other covariates in the model and the longitudinal relationship. In this report we multiply β1 by 10; hence, it measures the average 10-year change of a dependent variable during the follow-up. This model also simultaneously examines the cross-sectional relationship between each of the independent variables (e.g., BMI) and a dependent variable (e.g., SBP) and the relationship between changes in these independent variables and longitudinal changes in the dependent variable. Because cigarette smoking was uncommon among females, this covariate was not included in the model for females.

## Results

### Baseline characteristics of the cohorts

Characteristics of the four gender-occupation groups at baseline are shown in Table 1. The mean age of workers and farmers was similar for both males and females. The education levels were rather low and lower in farmers than in workers (*P *< 0.01). Smoking was very common among males but was uncommon among females. Farmers smoked more than workers within the same gender. Likewise alcohol drinking was much more common among males than females and more common among farmers than workers (*P *< 0.01).

**Table 1:** Baseline characteristics by gender and occupation among cohort populations in Guangzhou, China, 1983-1984

	Males				Females			
	**Workers**		**Farmers**		**Workers**		**Farmers**	

	**Mean or %**	**SD**	**Mean or %**	**SD**	**Mean or %**	**SD**	**Mean or %**	**SD**

Age (years)	42.1	8.7	42.4	8.9	41.1	8.2	41.7	9.6

Education (%)			*				*	

Never school	2.1		8.3		11.5		57.4	

Primary school	36.8		75.3		34.3		40.4	

Middle school	35.7		14.1		25.0		1.8	

High school or above	25.4		2.3		29.2		0.5	

Current smoker (%)	71.7		81.8*		2.8		5.3*	

Cigarettes per day								

Total	12.7	11.8	18.6*	14.8	0.2	1.8	0.5*	2.9

Current smoker	17.7	10.3	22.7*	13.2	8.4	7.2	9.7	8.2

Current alcohol drinker (%)	24.9		61.0*		1.8		7.7*	

Alcohol intake (g/per day)								

Total	10.6	28.9	51.8*	74.0	0.4	4.5	1.3*	7.3

Drinker	42.4	44.8	84.9*	78.5	20.4	27.4	17.3	20.5

Systolic blood pressure (mmHg)	115.0	14.8	115.5	14.0	114.4	17.2	109.8*	13.9

Diastolic blood pressure (mmHg)	74.6	9.8	72.3*	9.5	74.0	10.5	68.8*	9.1

Hypertension (%)	9.7		7.4*		10.5		3.8*	

Taking antihypertensive medication (%)								

Total	1.3		0.3*		1.3		0.3*	

Hypertensive	13.8		3.6*		12.1		6.7	

Body mass index (kg/m^2^)	20.5	2.4	19.9*	1.9	21.4	3.0	19.7*	2.0

Weight category (%)			*				*	

Normal	95.3		99.1		88.3		98.9	

Overweight	4.7		0.9		10.8		1.0	

Obesity	0.0		0.1		1.0		0.1	

Total cholesterol (mg/dl)	181.9	32.0	158.6*	30.3	187.2	33.2	155.4*	28.7

Low density cholesterol (mg/dl)	112.2	28.4	94.0*	25.3	114.5	29.2	92.4*	25.0

High density cholesterol (mg/dl)	49.6	12.1	49.1	12.2	53.4	12.4	47.9*	10.4

Triglycerides (mg/dl)	104.9	86.4	80.8*	77.6	100.3	90.5	76.1*	44.0

*P < 0.01 comparing workers and farmers within the same gender

Except for SBP among males, average blood pressure levels were higher among workers than farmers within the same gender (*P *< 0.01). Prevalence of hypertension was lower than 11% and likewise higher among workers than farmers (*P *< 0.01). Only a small percent of persons with hypertension were taking antihypertensive medication, especially among farmers.

Average BMI and prevalence of overweight (including obesity) were rather low (ranging from 1.0% to 11.8%) and higher among workers (especially female workers) than farmers (*P *< 0.01). Workers had significantly higher TC, Low density lipoprotein cholesterol (LDL-C), and triglycerides levels than did farmers (*P *< 0.01).

### Changes in the levels of cardiovascular risk factors during follow-ups

Table 2 presents the prevalence of current smoking, hypertension, overweight (including obesity), and hyperlipidemia at baseline (year 0) and during follow-ups (year 4 and year 10). The prevalence estimates and the average change (linear trends) in 10 years were derived by the GEE method adjusting for age and the correlations of repeated measures. Prevalence of current smoking decreased significantly in every gender-occupation group. However, at year 10, age-adjusted smoking rate was still very high among males (60.5% in workers and 72.0% in farmers). There was a significant increase in the prevalence of hypertension in all cohorts. Correspondingly, prevalence of antihypertensive medication use also increased. The 10-year average percentage point increase in medication use in the cohorts was 1.4 among male workers (p = 0.016), 0.6 among male farmers (p = 0.076), 3.6 among female workers (p < 0.001), and 0.4 among female farmers (p = 0.091). The percentage of persons with hypertension who reported taking antihypertensive medication increased significantly among workers but not farmers (data not shown). The treatment rate remained low at 10 years, i.e., 21.9% among male workers, 5.0% among male farmers, 28.6% among female workers, and 9.3% among female farmers. Although the prevalence of overweight (including obesity) remained low at follow-ups (e.g., 13.4% among male workers, 6.8% among male farmers, 18.4% among female workers, and 6.0% among female farmers at year 10), these represented significant increases above baseline. Likewise, age-adjusted prevalence of hyperlipidemia increased significantly in the four gender-occupation groups (except female workers).

**Table 2:** Age-adjusted prevalence of selected risk factors by year, gender and occupation among cohort populations in Guangzhou, China, 1983-1994

	Males		Females	
	**Workers**	**Farmers**	**Workers**	**Farmers**

Current smoking (%)				

Year 0	71.3	80.4	4.3	6.7

Year 4	64.3	75.1	3.3	4.3

Year 10	60.5	72.0	0.8	2.1

Average change in 10 years	-10.6*	-8.3*	-3.5*	-4.6*

Hypertension (%)				

Year 0	13.9	10.0	15.7	6.0

Year 4	9.0	11.9	11.6	5.9

Year 10	18.8	22.0	20.7	10.7

Average change in 10 years	+5.5*	+12.3*	+5.5*	+5.0*

Overweight or obesity (%)				

Year 0	5.8	0.8	14.2	1.4

Year 4	6.2	2.2	16.2	2.1

Year 10	13.4	6.8	18.4	6.0

Average change in 10 years	+7.8*	+6.1*	+4.2*	+4.8*

Hyperlipidemia (%)				

Year 0	6.7	1.7	13.0	2.7

Year 4	15.3	5.1	18.8	3.8

Year 10	14.0	8.6	16.1	5.2

Average change in 10 years	+6.2*	+6.8*	+2.3	+2.5*

Note: Hypertension was defined as SBP ≥ 140 mmHg and/or DBP ≥ 90 mmHg or on antihypertensive medication. Overweight was defined as body mass index ≥25 kg/m^2 ^and obese was defined as body mass index ≥30 kg/m^2^. Hyperlipidemia was defined as serum total cholesterol ≥240 mg/dl and/or low density lipoprotein cholesterol ≥160 mg/dl. *P < 0.001 for 10 years linear trend

We calculated the age-adjusted average 10-year change of risk factor levels (continuous variables) by the GEE method (Table 3). Average SBP and DBP increased significantly in all four groups with the biggest increase among male farmers. BMI also increased significantly and the biggest increase (1.4 kg/m^2 ^in 10 years) was among farmers. Cigarette smoking and alcohol drinking were uncommon in females. Average TC and LDL-C increased significantly, especially among farmers. High density lipoprotein cholesterol (HDL-C) changed little in workers but significantly increases in farmers. Average triglycerides markedly increased among male workers and male farmers.

**Table 3:** Age-adjusted average changes of risk factor levels by gender and occupation among cohort populations in Guangzhou, China, 1983-1994

	Male				Female			
	**Workers**		**Farmers**		**Workers**		**Farmers**	

	**β**	**SE**	**β**	**SE**	**β**	**SE**	**β**	**SE**

Systolic blood pressure (mmHg)	4.9**	0.6	7.6**	0.6	4.6**	0.6	4.2**	0.5

Diastolic blood pressure (mmHg)	2.6**	0.4	6.2**	0.4	1.0**	0.4	4.6**	0.3

Body mass index (kg/m^2^)	1.2**	0.1	1.4**	0.1	0.6**	0.1	1.4**	0.1

Cigarettes per day								

Total	-2.8**	0.4	-2.5**	0.6	^a^		^a^	

Current smoker	-1.5**	0.4	-1.2*	0.6	^a^		^a^	

Alcohol intake (g/per day)								

Total	-4.4**	1.2	-9.2**	2.6	-0.3*	0.1	-0.2	0.2

Drinker	-12.3**	3.0	-13.3**	3.5	-8.6*	4.2	-4.3	3.0

Total cholesterol (mg/dl)	9.1**	1.3	22.8**	1.4	3.1*	1.3	17.0**	1.1

Low density cholesterol (mg/dl)	6.5**	1.2	11.7**	1.3	3.0*	1.2	7.7**	1.0

High density cholesterol (mg/dl)	-0.8	0.6	7.0**	0.7	0.8	0.6	9.0**	0.5

Triglycerides (mg/dl)	20.6**	3.9	24.4**	4.5	-5.8	3.4	1.9	2.0

**P < 0.01; *P < 0.05; and ^a^Not estimated because few females smoked

### Factors associated with blood pressure and changes in blood pressure

Table 4 presents the results of the GEE analyses examining the independent longitudinal changes of SBP, factors associated with SBP, and changes of factors associated with change in SBP. The positive coefficients of time indicate that average SBP increased significantly in 10 years after adjustment for aging of the cohorts and other factors (including the changes of the factors) in the models. The large coefficients of the antihypertensive medication usage indicate that SBP in hypertensive persons was far from being control.

**Table 4:** Factors associated with systolic blood pressure and changes in systolic blood pressure by gender and occupation among cohort populations in Guangzhou, China, 1983-1994

	Males				Females			
	**Workers**		**Farmers**		**Workers**		**Farmers**	

	**β**	**SE**	**β**	**SE**	**β**	**SE**	**β**	**SE**

Time (10 years)	3.0**	0.7	5.5**	0.7	4.1**	0.7	2.7**	0.5

Age (year)	0.4**	0.1	0.2**	0.1	0.7**	0.1	0.5**	0.1

Education (level^a^)	-0.7*	0.4	0.2	0.6	-0.2	0.4	-0.01	0.5

Antihypertensive medication use	13.8**	1.9	15.9**	3.1	13.1**	1.7	17.3**	3.5

Body mass index (kg/m^2^)	1.5**	0.1	1.0**	0.2	1.5**	0.1	0.6**	0.1

Change in body mass index (kg/m^2^)	1.5**	0.2	1.5**	0.3	1.1**	0.2	1.0**	0.1

Cigarettes per day (10/d)	-1.6**	0.3	-1.2**	0.2	^b^		^b^	

Change in cigarettes per day (10/d)	0.7*	0.4	0.8*	0.3	^b^		^b^	

Alcohol intake (13.7 g/d^c^)	0.5**	0.2	0.3**	0.1	-0.7	0.7	-1.0	0.4

Change in alcohol intake (13.7 g/d^c^)	-0.3	0.2	-0.4**	0.1	0.2	1.0	0.9	0.5

**P < 0.01; *P < 0.05; ^a^Four education levels: never attended school, primary school, middle school, and high school or higher; ^b^Not included in the model because few females smoked; and ^c^13.7 g/d is equivalent to 1 drink/d

Baseline BMI was significantly and directly associated with SBP, and increase in BMI was significantly associated with increase in SBP in every gender-occupation group (Table 4). Although cigarette smoking was inversely associated with average SBP, increase in the amount of smoking was associated with increase in SBP among males. Baseline alcohol intake was significantly and directly associated with SBP among males. Except among male farmers, change in the amount of drinking was not associated with change in SBP.

Overall, the results of GEE analyses for DBP were similar to those of SBP.

### Factors associated with serum lipids and changes in serum lipids

Table 5 presents the results of GEE analyses for TC. Except among female workers, the regression coefficient of time was statistically significant, indicating a significant increase in TC over time. Baseline BMI was directly related to average TC and increase in BMI was related to increase in TC among all four groups. Cigarette smoking was not significantly related to average TC, but increase in the amount of smoking was directly related to increase in TC among male farmers. Among males, baseline alcohol intake was directly related to average TC and increase in the amount of intake was positively related to increase in TC.

**Table 5:** Factors associated with serum total cholesterol and changes in serum total cholesterol by gender and occupation among cohort populations in Guangzhou, China, 1983-1994

	Males				Females			
	**Workers**		**Farmers**		**Workers**		**Farmers**	

	**β**	**SE**	**β**	**SE**	**β**	**SE**	**β**	**SE**

Time (10 years)	5.4**	1.4	19.4**	1.6	2.0	1.5	13.2**	1.2

Age (year)	0.8**	0.1	-0.1	0.1	1.8**	0.1	1.2**	0.1

Education (level^a^)	3.4**	1.0	1.4	1.6	0.9	0.8	4.3**	1.3

Body mass index (kg/m^2^)	1.4**	0.4	1.4**	0.4	1.2**	0.3	1.5**	0.4

Change in body mass index (kg/m^2^)	2.3**	0.5	3.1**	0.6	1.3**	0.4	1.8**	0.3

Cigarettes per day (10/d)	-0.2	0.8	0.6	0.6	^b^		^b^	

Change in cigarettes per day (10/d)	-0.6	0.6	1.0*	0.5	^b^		^b^	

Alcohol intake (13.7 g/d)	1.3**	0.5	0.9**	0.2	1.8	2.2	-0.1	0.9

Change in alcohol intake (13.7 g/d)	0.6*	0.3	0.4*	0.2	0.9	1.1	0.7	1.1

**P < 0.01; *P < 0.05; ^a^Four education levels: never attended school, primary school, middle school, and high school or higher; ^b^Not included in the model because few females smoked; and ^c^13.7 g/d is equivalent to 1 drink/d

For other lipid measures, i.e., LDL-C, HDL-C, and triglycerides, the multivariate-adjusted regression coefficients of time were statistically significant among most gender-occupation groups (except LDL-C in female workers and triglycerides in either female workers or female farmers) (data not shown). The associations between baseline BMI and the three lipid measures were all statistically significant (directly for LDL-C, triglycerides; and inversely for HDL-C) among every gender-occupation group. Likewise, change in BMI was significantly and directly associated with change in LDL-C, triglycerides; and inversely associated with change in HDL-C.

### Additional analysis

We repeated the analyses assuming that the mean TC were 2.2 mg/dl higher than they would have been had all the 1993-1994 measurements been made using fresh sera. The age-adjusted average 10-year increase in TC was 6.7 mg/dl for male workers, 20.6 mg/dl for male farmers, 0.8 mg/dl for female workers and 14.7 mg/dl for female farmers. The coefficients for follow-up time were statistically significant among all groups except female workers in GEE analyses. The relations between baseline measures of covariates and the average TC and between the changes in covariates and changes in TC were similar to those without correction for the individual TC values (data not shown).

## Discussion

This cohort study of workers and farmers in southern China shows significant adverse trends of cardiovascular profiles in a 10 years period independent from the aging process of the cohorts. The results of this study confirm the rapid epidemiologic change underway in these populations that used to have very low cardiovascular risk.

Most of the studies evaluating changes in the levels of cardiovascular risk factors in the population were conducted through multiple cross-sectional observations on non-overlapping samples [6,8,19,20]. The limitation of such a design is that differential cohort effects in the different cross-sectional samples may bias the estimate [21]. Differing pre-baseline experiences among samples from different cross-sections (i.e., cohort or generation effect) is likely to be large in China, with dramatic political, economic, and societal changes in a relative short time. This study used repeated measures of the same individuals to examine the time trends, independent from biologic aging, and have examined the factors related to these trends. The current cohort study is also unique in having small numbers of people who were taking antihypertensive medication or lipid-lowering medication during the study period. Thus, it is possible to assess the true levels and trends of blood pressure and lipids in the populations.

The associations between increasing BMI and increasing blood pressure and lipids suggest that for prevention of the age-related rise in blood pressure and lipid levels, and ultimately of hypertension and hyperlipidemia, it is important to prevent weight gain, even among the lean populations, when blood pressure and lipids levels are still low. A survey in 2002 in Guangdong Province, where Guangzhou is located, reported that the prevalence of overweight or obesity in adult residents was 28.0% among urban males, 9.9% among rural males, 22.6% among urban females, and 9.7% among rural females [22]. Although these prevalences were much lower than those in Western populations, they were higher than those in our cohorts in 1993-1994, i.e., 13.4% among male workers, 6.8% among male farmers, 18.4% among female workers, and 6.0% among female farmers.

Increasing body weight in the populations accounted for part of the observed changes in risk factor profile among these cohorts. Other factors not measured directly may also contribute to the changes, including the change in body weight. China is undergoing a remarkably fast, yet undesirable, nutritional transition dominated by increasing intake of fat and animal foods [23]. The classic diet in Guangzhou contained high complex carbohydrates and dietary fiber (e.g., cereals, starchy roots), fish, fruits, and vegetables, with few meat and animal foods [24]. Three-day dietary recalls on subsamples of our study cohorts were collected in 255 participants at baseline and 203 participants in the second re-examination. The average Key score [25]--a measure of intake of saturated fatty acids relative to polyunsaturated fatty acids, as well as the intake of dietary cholesterol - increased among all four gender-occupation groups (a 25% increase overall) [9].

The economy of Guangzhou has changed greatly during the study period. In both urban and rural areas, inflation-adjusted personal income increased five-fold during 1984-1993 [9]. Prosperous economic growth, commercialization, and availability of foods resulted in more people eating out or eating out more often. The increasing pace of work/and life and time constraints may force people to "eat on the go". China is no longer a "bicycle country". Bike use dropped from 34% of total trips to 24% in one decade from 1980s to 1990s in Guangzhou [26]. In contrast, motorized trips increased, including public transport, passenger cars, taxi, or motorcycle. Uncontrolled growth of urban sprawl has led to long commutes for workers by buses or trolleybus, passenger cars, or subways [27]. Urbanization and commercialization have resulted in a more sedentary lifestyle and less work-related or leisure time physical activities.

Despite a favorable decreasing trend, smoking remained very common among males at year 10 (60.5% among male workers and 72.0% among male farmers). China is the world's largest cigarette consumer and producer [28]. It's state-owned tobacco monopoly generated profit and taxes that were 11% of the central government's total revenue in 1996 [28]. The tobacco control policy is heavily influenced by economic rather than public health policy. Strong tobacco control efforts are urgently needed in China.

Major cardiovascular risk factors were similar in Western and Chinese populations [29]. This has important implications given the enormous sizes of the populations in China. Blood pressure is strongly and directly related to vascular and overall health, without any evidence of a threshold down to at least 115/75 mmHg [30]. Mass population interventions that use dietary, lifestyle, or environmental factors to move blood pressure distribution downwards has the potential to produce large reductions in the burden of cardiovascular disease in China [31]. A reduction in the average DBP of just 3 mmHg would avert approximately one-third of stroke deaths in China, where stroke is more common than coronary heart disease [32]. In addition, data showed that there is no threshold below which a lower TC concentration (e.g., below 154 mg/dl, 4 mmol/l) is not associated with a lower risk of ischemic heart disease [33]. The sharp increase in serum lipids observed among workers and farmers is alarming. Data from Eastern Europe suggested that cholesterol level responded rapidly to changes in lifestyle and diet, and that mortality increased relatively quickly after the cholesterol increases [34]. In addition, the absolute excess risks for higher BMI and smoking were additive [35]. Induction periods from exposure to a risk factor to a disease occurrence may be shorter when other causal factors are common [36]. In Guangzhou, smoking was already very prevalent (in men) before blood pressure and cholesterol levels increased. Therefore, a sharp increase in prevalence, incidence, and mortality of cardiovascular disease can be predicted.

This study has several limitations. The participants were recruited from cluster samples from one factory and one rural area and were aged 25-64 years at baseline. They were not the representative samples of the general populations. In addition, there was a potential healthy worker effect of the cohorts. The follow-up examinations were not performed after 1994. China has seen remarkable economic growth over the past 30 years. It is expected that large changes in risk factor levels continued beyond 1994 that were not captured in this report.

## Conclusions

This cohort study of low risk workers and farmers in south China indicates unfavorable trends of cardiovascular risk factors from 1983-1994. Increase in BMI was significantly associated with increase in blood pressure and TC levels. The unfavorable trends persisted after adjusting for aging of the cohort, education, BMI, smoking, and alcohol intake. The adverse changes in risk factor levels mirrored the dramatic economic and environment changes in the society. Thus, in addition to changes in the behavior, attitudes, and social values of individuals, change to society, systems, and policies that promote major risk factors are crucial. Grass root primordial prevention is important and urgently needed to preserve these so far low risk populations from the penetration of risk factors and disease epidemics [37].

## Competing interests

The authors declare that they have no competing interests.

## Authors' contributions

XL participated in the design of the study, data analysis, and drafted the manuscript. JM participated in data analysis, interpretation of data, and manuscript revision. XR participated in the conception and design of the study, initiated the data collection, and manuscript revision.

QZ oversaw laboratory tests, quality control, and manuscript revision. CG, XG, YW, and MD assisted with data collection, study coordination, quality control, interpretation of the findings, and manuscript revision. SL conceived of the study, directed all stages of the study, participated in its design and coordination, and manuscript revision. All authors read and approved the final manuscript.

## Pre-publication history

The pre-publication history for this paper can be accessed here:

http://www.biomedcentral.com/1471-2458/11/931/prepub
